# Construction of a synthetic pathway for the production of 1,3-propanediol from glucose

**DOI:** 10.1038/s41598-019-48091-7

**Published:** 2019-08-09

**Authors:** Cláudio J. R. Frazão, Débora Trichez, Hélène Serrano-Bataille, Adilia Dagkesamanskaia, Christopher M. Topham, Thomas Walther, Jean Marie François

**Affiliations:** 1 Toulouse Biotechnology Institute (TBI), Université de Toulouse, CNRS, INRA, INSA, 135 Avenue de Rangueil, F-31077 Toulouse, France; 2 Molecular Forces Consulting, 40 rue Boyssonne, F-31400 Toulouse, France; 3 TWB, 3 Rue des Satellites, Canal Biotech Building 2, F-31400 Toulouse, France; 4 Present Address: TU Dresden, Institute of Natural Materials Technology, 01062 Dresden, Germany

**Keywords:** Biotechnology, Metabolic engineering

## Abstract

In this work, we describe the construction of a synthetic metabolic pathway enabling direct biosynthesis of 1,3-propanediol (PDO) from glucose via the Krebs cycle intermediate malate. This non-natural pathway extends a previously published synthetic pathway for the synthesis of (L)-2,4-dihydroxybutyrate (L-DHB) from malate by three additional reaction steps catalyzed respectively, by a DHB dehydrogenase, a 2-keto-4-hydroxybutyrate (OHB) dehydrogenase and a PDO oxidoreductase. Screening and structure-guided protein engineering provided a (L)-DHB dehydrogenase from the membrane-associated (L)-lactate dehydrogenase of *E*. *coli* and OHB decarboxylase variants derived from the branched-chain keto-acid decarboxylase encoded by *kdcA* from *Lactococcus lactis* or pyruvate decarboxylase from *Zymomonas mobilis*. The simultaneous overexpression of the genes encoding these enzymes together with the endogenous *ydhD*-encoded aldehyde reductase enabled PDO biosynthesis from (L)-DHB. While the simultaneous expression of the six enzymatic activities in a single engineered *E*. *coli* strain resulted in a low production of 0.1 mM PDO from 110 mM glucose, a 40-fold increased PDO titer was obtained by co-cultivation of an *E*. *coli* strain expressing the malate-DHB pathway with another strain harboring the DHB-to-PDO pathway.

## Introduction

A central goal of the bioeconomy consists in reducing our dependence on petroleum by focusing on the development of efficient, sustainable and eco-friendly processes for production of bio-based chemicals and fuels^1,2^. 1,3-Propanediol (PDO) is an important commodity chemical that can serve as a precursor monomer for the synthesis of industrially relevant polymers, including polyesters, polyethers and polyurethanes. Among those, the polyester polytrymethylene terephthalate (PTT) is foreseen as a major competitor of Nylon in carpet industries, therefore making PDO a molecule of significant industrial interest. While both acrolein hydration, and ethylene oxide hydroformylation followed by reduction of resulting aldehyde enable PDO synthesis, low product yields and harsh reaction conditions impede the successful development of a cost-competitive chemical production process^3–5^.

**Figure 1:**
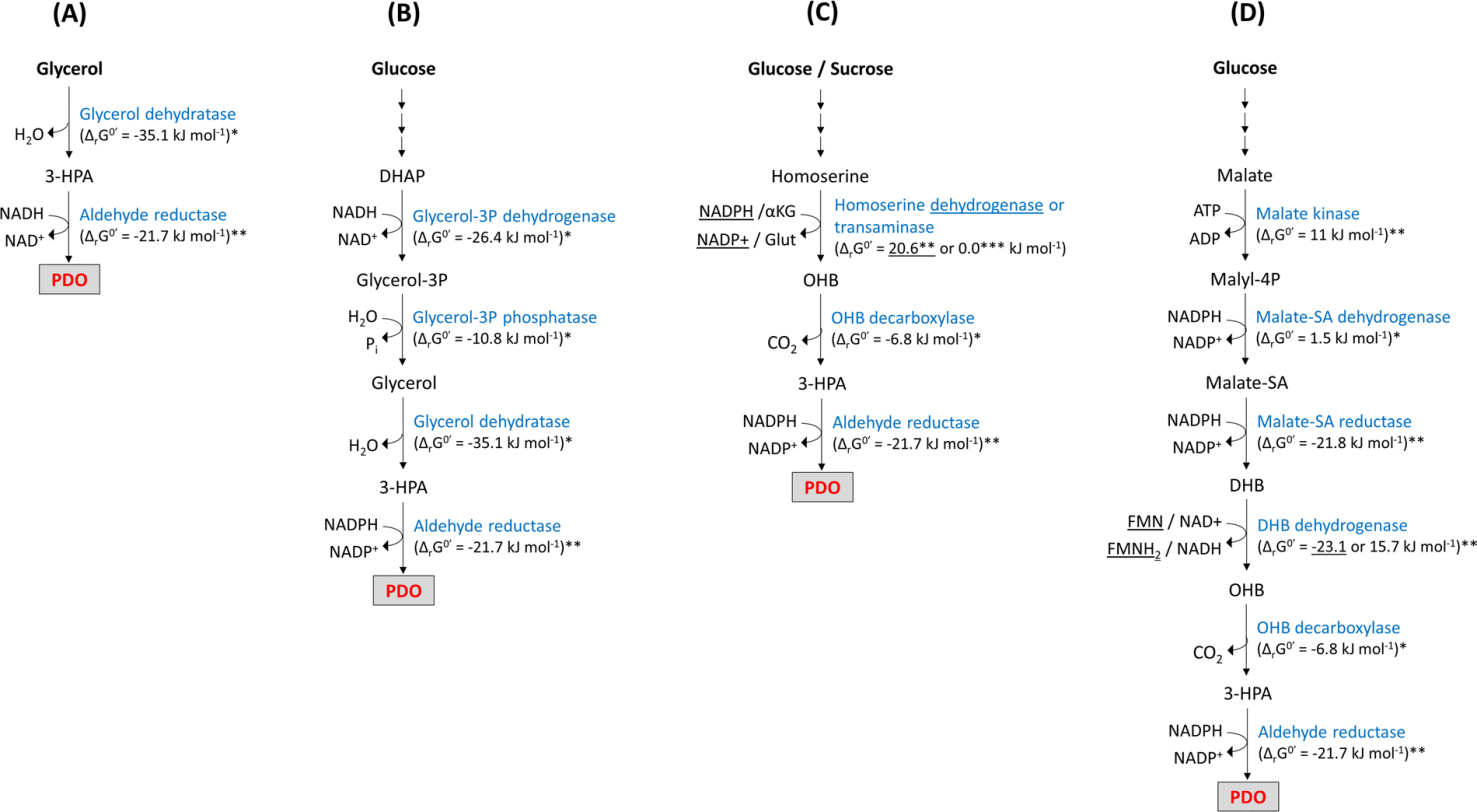
Natural (**A**) and engineered metabolic pathways (**B**–**D**) enabling biosynthesis of 1,3-propanediol (PDO). The standard Gibbs free energy values of each individual reaction was taken from EcoCyc database when available (denoted as *). Otherwise, values were estimated based on the group contribution theory^43^ (denoted as **).

Alternatively, PDO can be produced from glycerol by *Klebsiella*, *Clostridium*, *Citrobacter*, *Enterobacter* or *Lactobacillus* species under anaerobic conditions in a two-step reductive pathway that employs vitamin B_12_-dependent glycerol dehydratase and PDO oxidoreductase enzymatic activities^5^. More recently, the occurrence of a vitamin B_12_-independent glycerol dehydratase in *C*. *butyricum* (Cb-DhaB1) has also been demonstrated and employed for PDO biosynthesis^6^. PDO biosynthesis from glycerol has previously been shown to be extremely efficient. For instance, batch cultivation of *K*. *pneumoniae* enabled the production of up to 60 g L^−1^ PDO at a high formation rate (1.4 g L^−1^ h^−1^) and good yield (0.35 g PDO per g glycerol)^3^. A particularity of the PDO pathway starting from glycerol is that product yield remains dependent on the intracellular availability of the NADH co-factor. While anaerobic cell growth on glycerol (oxidative pathway) ensures NADH formation, co-factor regeneration needs the additional formation of a by-product to serve as electron sink (e.g. acetate, butyrate, ethanol, butanol). Together with the fact that glycerol dehydratases are inhibited by the presence of substrate/product at elevated concentration, titre and production rates of PDO to be achieved are limited unless newly discovered or engineered enzymes are found to be less sensitive to the substrate/product couple^7,8^. In attempt to demonstrate additional entry points for PDO production and overcome the potential challenges imposed by glycerol fermentation, various strategies were developed to synthesize PDO from sugar feedstock. While no natural microorganisms have been found to directly convert sugars to PDO, the adoption of two-stage and co-fermentation processes was shown to enable a better control of cultivation conditions and a wider flexibility in terms of substrate utilization^5,9,10^. Another approach is the engineering of microorganisms for the direct production of PDO from sugars via glycerol, which led Genencor and DuPont to develop a fermentation process employing a genetically modified *E*. *coli* strain able to produce PDO aerobically at high titer and yield^11^. In both cases, the engineered PDO pathways are composed of the necessary reaction steps linking the glycolysis-derived metabolite dihydroxyacetone phosphate (DHAP) to PDO via glycerol (Fig. 1B), thereby increasing the theoretical product yield by 50% (1.5 mol PDO per mol glucose) when compared to the utilization of glycerol as carbon source. While in the last decade glycerol costs dropped considerably reaching prices which today are inferior to that of glucose, the main advantage of glucose-based processes is the substantially increased product titers and rates as in theory glycerol is not accumulated at high-titers and glycerol dehydratases become no longer rate-limiting^12^.

Traditional metabolic engineering resulted in great improvements in PDO biosynthesis, but the rapid development of powerful genome mining and protein engineering tools enable now the creation of *de novo* designed non-natural pathways for the production of economically relevant compounds^13^. In this regard, an artificial pathway enabling PDO to be synthesized from glucose or sucrose was previously implemented in *E*.*coli* by extending the naturally occurring aspartate-homoserine pathway (Fig. 1C). The proposed pathway has a similar theoretical product yield than that of Dupont’ route and does neither involve glycerol as intermediate nor the utilization of glycerol dehydratase enzymes, and is therefore very appealing for PDO production. This approach, which has been patented by METEX^14^, involves an enzyme bearing amino transferase activity on homoserine, followed by the decarboxylation of 2-keto-4-hydroxybutyrate (OHB) by a keto-acid decarboxylase, and finally the reduction of hydroxypropanal (3-HPA) to PDO by an enzyme with 1,3-propanediol oxidoreductase activity. While the last reaction of this artificial pathway is catalyzed by the broad substrate range NADPH-dependent aldehyde reductase encoded by *yqhD* gene from *E*. *coli* (Ec-*yqhD*)^15^, the two preceding enzymatic reactions are not known to naturally occur in cell metabolism. In demonstration of this pathway, these authors have engineered an *E*. *coli* strain overexpressing the native *serC*-encoded transaminase (Ec-SerC) and the branched-chain 2-ketoisovalerate decarboxylase from *Lactococcus lactis* (Ll-KivD)^16^. In a complementary study, the poorly effective homoserine transaminase in this pathway has been replaced by a homoserine dehydrogenase obtained by rational engineering of either an *E*. *coli* NADP-dependent glutamate dehydrogenase^17^ or Ec-SerC^18^. However, PDO titer obtained with these engineered enzymes was still in the range of mg per liter, suggesting that the deamination of homoserine is rate limiting in the pathway.

To overcome this problem and propose an new alternative pathway for PDO synthesis from glucose, we took advantage of our recent synthetic pathway leading to the production the non-natural metabolite (L)-2,4-dihydroxybutyrate (L-DHB) that departs from the TCA cycle intermediate malate through three non-natural enzymatic reaction steps^19^. As described in Fig. 1D, malate is converted to malyl-phosphate by employing malate kinase, followed by a malate semialdehyde dehydrogenase and then malate semialdehyde reductase activities to yield L-DHB. Extending this pathway with (L)-DHB dehydrogenase, OHB decarboxylase and PDO oxidoreductase shall eventually produce PDO. Our proposed pathway has a theoretical product yield identical to other PDO pathways starting from glucose (1.5 mol per mol glucose), and consumes 1 mol of ATP and 3 mol of NADPH per consumed mol of malate, being well suited for aerobic based-processes. Based on enzyme screening and engineering approaches, the required enzyme activities were improved and found to enable *in vivo* PDO production from DHB. While simultaneous expression of all six enzymatic activities in one *E*. *coli* strain enabled direct PDO production from glucose, distributing the malate-to-DHB and the DHB-to-PDO pathway modules into two *E*. *coli* strains co-cultivated in mineral medium further improved PDO titer.

## Results

### Design of the PDO synthetic pathway

In a previous study, we designed and experimentally validated a *de novo* metabolic pathway leading to the production of (L)-DHB from glucose via malate by expressing malate kinase, malate semialdehyde dehydrogenase and malate semialdehyde reductase enzyme activities in *E*. *coli*^19^. While DHB can be transformed to the methionine-analog HMTB by chemical means^20^, we herein demonstrate that this non-natural metabolite may also serve as a precursor for the microbial production of PDO. The conversion of (L)-DHB to PDO proceeds via three reaction steps (Fig. 1D): (L)-DHB is first oxidized to OHB, the resulting α-keto acid is then decarboxylated to yield 3-HPA, which is finally reduced into PDO. These reactions are catalyzed by enzymes bearing (L)-DHB dehydrogenase, OHB decarboxylase and PDO oxidoreductase activities, respectively. The negative standard Gibbs free energy for the proposed pathway attests its thermodynamic feasibility (Table S1 and Note 1 in Supplementary Information). Stoichiometric analysis of the metabolic network in *E*. *coli* shows that PDO can be produced from glucose with a theoretical maximum yield of 1.5 mol mol^−1^ in the absence of cell growth (Fig. S1 and Note 2 in Supplementary information). The theoretical yield of PDO is similar to those from glucose via homoserine^17^ and via glycerol^11^. Implementation of the pathway requires enzymes bearing the three constitutive enzymatic activities. The promiscuous broad range aldehyde reductase encoded by *yqhD* in *E*. *coli* was previously shown to catalyze the reduction of 3-HPA to PDO Jarboe, 2011 #9565}, and was therefore used in our work. The *Zymomonas mobilis* pyruvate decarboxylase was found to exhibit OHB decarboxylase activity^17^, while to the best of our knowledge no natural (L)-DHB dehydrogenase activity has been reported to date.

### Engineering of (L)-DHB dehydrogenase activity

In a recent work, we showed that the soluble NAD-dependent (L)-malate dehydrogenase enzyme variant from *E*. *coli* Ec-Mdh-5Q (I12V:R81Q:M85Q:D86S:G179D) was able to oxidize DHB into OHB as substrate^21^. However, the unfavorable thermodynamics of NAD-dependent DHB oxidation (Δ_r_G^0^ = 15.7 kJ mol^−1^) together with the low affinity of this enzyme variant for (D/L)-DHB (K_m_ ~ 130 mM) prompted us to screen for alternative DHB dehydrogenase candidate enzymes. Remarkably, *E*. *coli* MG1655 wild-type cells were found to possess the capability to assimilate 20% of 50 mM (D/L)-DHB after 24h of cultivation in mineral medium (data not shown). The identification of enzymes responsible for this assimilation of DHB dehydrogenase was therefore investigated. In particular, *E*. *coli* displays a set of three lactate dehydrogenase enzymes able to catalyze the interconversion between L- and D-lactate and pyruvate encoded by *ldhA*, *llDd*, *dld* genes. The Ec-LldD and LlldD enzyme relies on FMN and FAD-dependent co-factor system, respectively^22,23^, which allow the oxidation of lactate (Δ_r_G^0^ = −23.1 kJ mol^−1^; Note 2 in Supplementary information). Since the deletion of the corresponding *lldD* gene in *E*. *coli* nearly abolished DHB consumption (less than 1% consumption after 24h of cell cultivation, not shown), we decided to further characterize the Ec-LldD enzyme as a putative DHB dehydrogenase. To this end, the wild-type Ec-LldD enzyme was produced from a pET28-derived vector expressed in *E*. *coli* BL21 (DE3). As LldD is a membrane-associated enzyme and hence hardly to purify to homogeneity, we evaluated V^max^ and K_M_ of this enzyme directly on in crude protein extracts owing to the fact that the measured activity of this enzyme was highly reproducible from independent cultures expressing this gene in pET28^+^. While LldD activity on DHB and on (L)-lactate in cells bearing an empty plasmid was barely detectable (V^max^ < 0.01 U mg^−1^; Table 1), overexpression of the gene encoding this enzyme resulted in a more than 50 fold increase of activity on these two substrates. However, the activity as well as the affinity of LldD on DHB were respectively 4 and 250 fold lower than on L-lactate (Table 1). Although these data were obtained on crude extracts and therefore does not provide true kinetic constant (k_cat_) and catalytic efficiency (k_cat_/K_M_), they clearly justify for a rational engineering of LldD with the expectation to increase the affinity of this enzyme towards DHB.

**Table 1:** Membrane-associated 2-hydroxyacid dehydrogenase activities of *E*. *coli* BL21(DE3) cells expressing candidate his-tagged DHB dehydrogenases from the high-copy pET28 plasmid.

Plasmid	(L)-lactate		(D/L)-2,4-dihydroxybutyrate	
	V^max^ [U mg^−1^]^a^	K_M_ [10^−3^ M]	V^max^ [U mg^−1^]^a^	K_M_ [10^−3^ M]
pET28	0.01 (±0.000)^c^	nd	0.01 (±0.0005)^c^	—
pET28-Ec-LlDd	2.1 (±0.2)	0.04 (±0.01)	0.6 (±0.1)	10.6 (±0.7)
pET28-Ec-LlDd_V108C_	3.0 (±0.5)	0.2 (±0.0)	0.5 (±0.1)	6.6 (±0.8)

Data presented as the mean (±S.D.) of at least two replicates.

^a^Activities were estimated in U mg^−1^ crude protein extract.

^b^Specificity corresponds to the ratio of (v_max_/K_m_) between the natural substrate (L)-lactate and (D/L)-DHB.

^c^Since no saturation was observed, activities were measured in the presence of either 50 mM (L)-lactate or 100 mM (D/L)-DHB.

nd: not determined.

**Figure 2:**
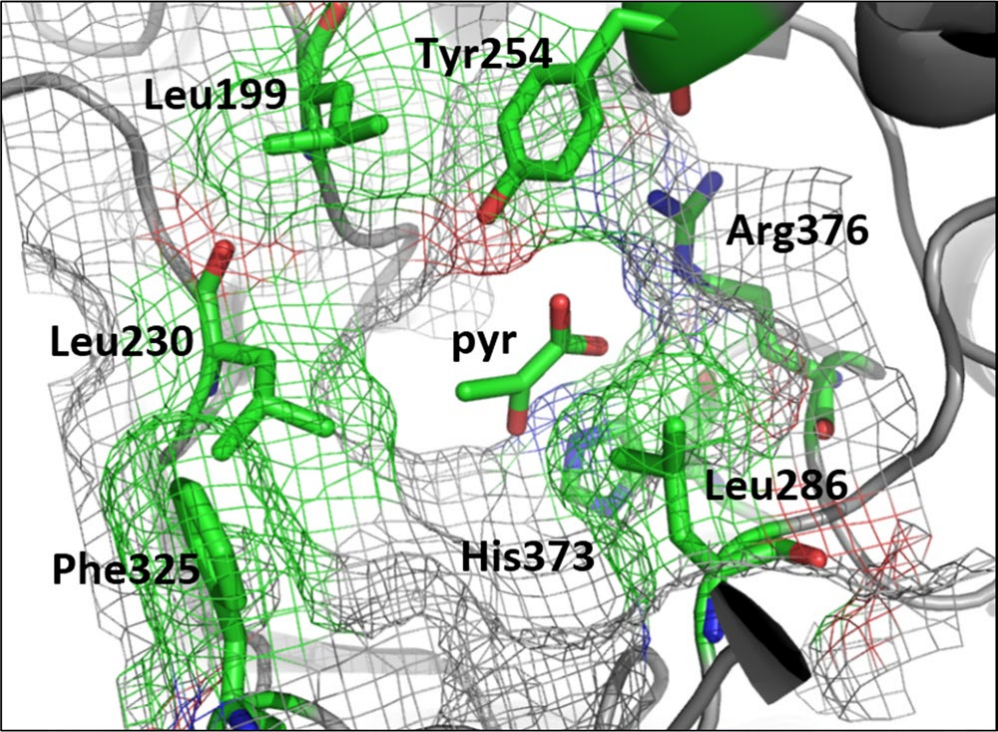
Active site region in the X-ray crystal structure of *S*. *cerevisiae* flavocytochrome B2 (Sc-Cyb2) (PDB code 1fcb). The residues in contact with reaction product pyruvate (pyr) are highlighted.

Amino acid residues responsible for substrate specificity in Ec-LldD were identified by visual inspection of the crystal structure of the homologous protein from *Saccharomyces cerevisiae* (Sc-Cyb2) bound to the reaction product pyruvate and the co-factor FMN^24^. Key residues in the active site of Sc-Cyb2 are shown in Fig. 2. The negative charge of the α-carboxylate group of pyruvate is neutralized by electrostatic interaction with Arg376, while residues Leu199, Leu230, Leu286, Tyr254 and Phe325 delimit the active site pocket. The catalytic residue His373 deprotonates the α-hydroxyl group of (L)-lactate, but the mechanism of substrate oxidation is still not clear. The pivotal role of those residues in substrate binding was previously discussed^25^. In the same study, amino acid substitution at residue Leu230 (corresponding to Val108 in Ec-LldD), which is in contact with the methyl group of pyruvate, was demonstrated to confer activity on larger substrates (e.g. mandelate). We therefore replaced valine in position 108 by smaller amino acid residues such as alanine, serine, cysteine and glycine, and analyzed the activities of resulting mutants (data not shown). Only the V108C variant resulted in an improved affinity for DHB (K_m_ = 6.6 mM) when compared to the wild-type enzyme (K_m_ = 10.6 mM; Table 1). While the increase in affinity towards the synthetic substrate was marginal, a decrease over 2-orders of magnitude in affinity towards (L)-lactate was observed.

### Engineering of OHB decarboxylase activity

The enzymes Zm-Pdc and the branched-chain ketoacid decarboxylase from *L*. *lactis* (Ll-KdcA) have been reported to possess activity on various 2-ketoacids, yielding corresponding aldehydes and carbon dioxide as reaction products^24^. Due to the structural similarity between OHB and 2-ketoacids, we therefore examined activities of these two enzymes on OHB. Results in Table 3 shows that both enzymes displayed OHB decarboxylase activities. However, as expected, they have a preference of up to 3-orders of magnitude for their natural substrate as compared to OHB. We therefore sought to engineer these two α-keto acid decarboxylase enzymes for higher catalytic efficiency towards OHB substrate by site-directed mutagenesis.

**Table 2:** Kinetic parameters of candidate OHB decarboxylases on 2-keto-4-hydroxybutyrate (OHB) and corresponding natural substrates.

Enzyme	Natural substrate^a^			2-keto-4-hydroxybutyrate				Specificity^c^
	V^max^ [U mg^−1^]	K_M_ [10^−3^ M]	V^max^/K_M_ [U mg^−1^ M^−1^]^b^	V^max^ [U mg^−1^]	K_M_ [10^−3^ M]	K_i_ [10^−3^ M]	V^max^/K_m_M [U mg^−1^ M^−1^]^b^	
Zm-Pdc	49.56 (±1.20)	1.7 (±0.2)	29,416 (±4,625)	0.01 (±0.0003)	0.4 (±0.2)	—	34 (±13)	864
Zm-Pdc W392Q	1.74 (±0.32)	5.2 (±0.3)	335 (±83)	0.02 (±0.004)	0.5 (±0.2)	5.0 (±0.4)	46 (±23)	7
Ll-KdcA	2.66 (±0.17)	2.7 (±0.2)	416 (±43)	0.02 (±0.001)	2.5 (±1.6)	—	9 (±5)	48
Ll-KdcA V461I	2.89 (±0.52)	5.1 (±1.1)	590 (±233)	0.04 (±0.0001)	1.3 (±0.5)	—	31 (±12)	19

Data presented are the mean (±S.D.) of at least two replicates.

^a^Natural substrates were used as follows: pyruvate for Zm-Pdc, 3-methyl-2-ketobutyric acid for Ll-KdcA.

^b^Activities were estimated in U mg^−1^ purified protein.

^c^Specificity corresponds to the ratio of (v_max_/K_m_) between natural substrate and OHB.

**Table 3:** Production of PDO from (D/L)-DHB by *E*. *coli* MG1655 wild-type (wt) and derived host strains harboring PDO downstream pathway.

Strain	Genes expressed	(D/L)-DHB consumed [mM]	(L)-DHB consumed [mM]	PDO produced [mM]	PDO conversion from L-DHB [%]
Pen946	pACT3	9.9 (±2.8)	9.4 (±2.9)	0.0 (±0.0)	0
Pen911	pACT3-llDd_V108C_-kdcA-yqhD	11.9 (±1.2)	11.9 (±1.2)	2.0 (±0.4)	17
Pen913	pACT3-llDd_V108C_-kdcA_V461I_-yqhD	11.9 (±1.9)	9.9 (±1.0)	5.5 (±0.5)	55
Pen965	pACT3-llDd_V108C_-pdc-yqhD	11.6 (±4.2)	10.8 (±4.4)	1.1 (±0.5)	11
Pen966	pACT3-llDd_V108C_-pdc_W392Q_-yqhD	11.1 (±0.04)	6.4 (±0.7)	7.1 (±0.3)	110

Cells were cultivated in 250 mL non-baffled shake flasks on mineral medium containing 20 g L^−1^ glucose. At OD_600_ ~0.6, IPTG (1 mM) and a racemic mixture of (D/L)-DHB (50 mM) were added to the medium. After 24 h of cell cultivation, PDO and (D/L)-DHB titers were measured. Reported data are the mean (±SD) of three independent replicates.

**Figure 3:**
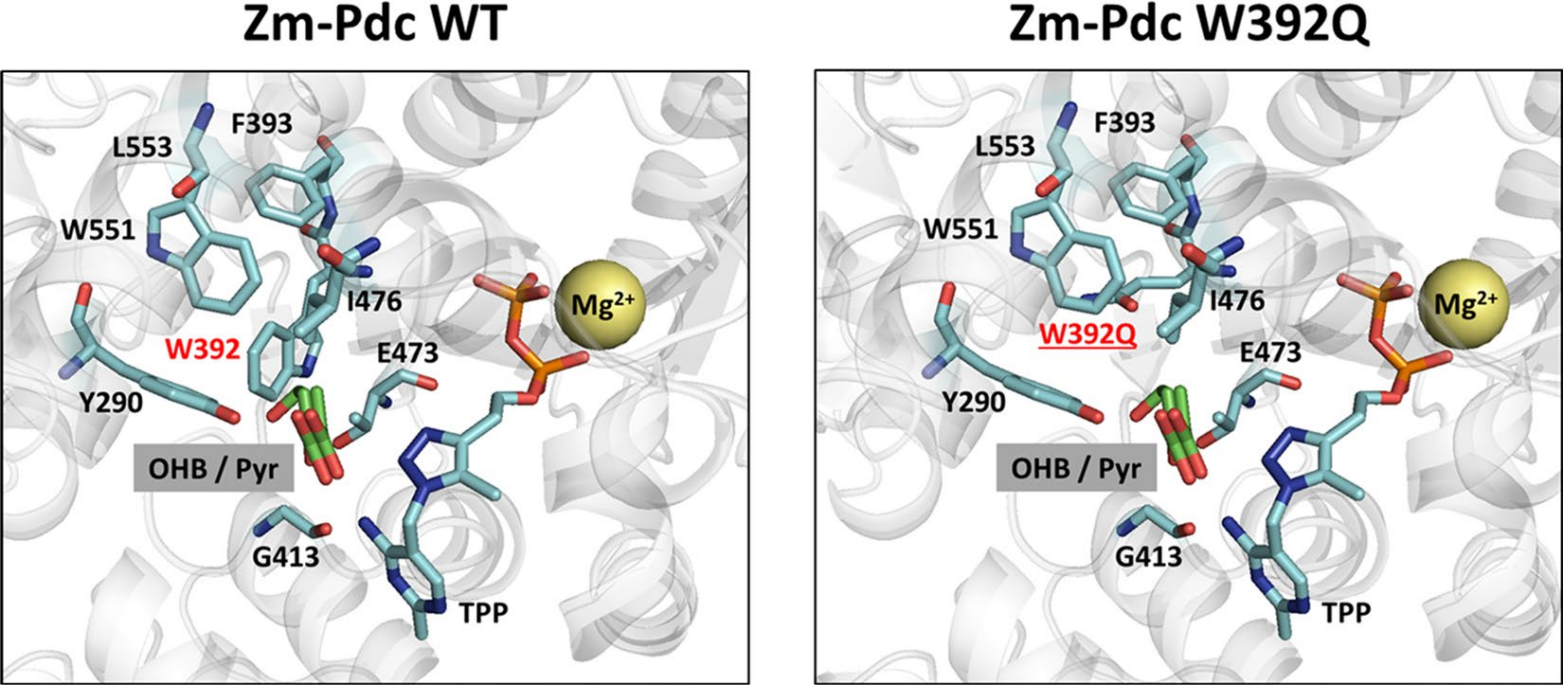
Active site region in the crystal structure of *Z*. *mobilis* pyruvate decarboxylase (Zm-Pdc) wild-type (**A**) and W392Q (**B**). The synthetic 2-keto-4 hydroxybutyrate (OHB) substrate was manually docked into the X-ray structure of the enzyme with bound pyruvate (Pyr) and a TPP analogue complexed with a Mg^2+^ ion (PDB code 2wva). The individual mutation at residue 392 was introduced to Zm-Pdc manually in Pymol and resulting change in pocket size is shown.

**Figure 4:**
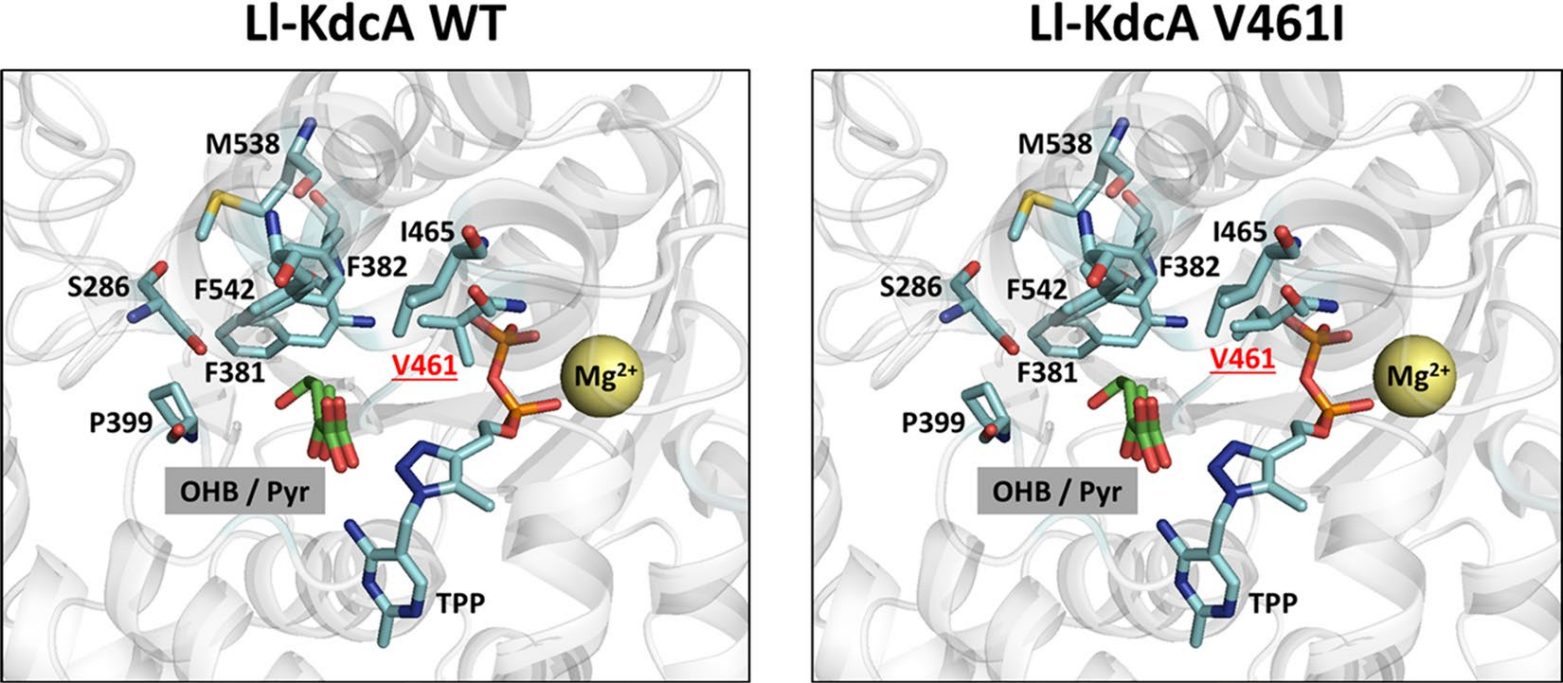
Active site region in the crystal structure of *L*. *lactis* branched chain keto-acid decarboxylase (Ll-KdcA) wild-type (**A**) and V461I (**B**). The X-ray structure bound with a TPP analogue complexed with a Mg^2+^ ion (PDB code 2vbg) was aligned with Zm-Pdc structure (PDB code 2wva) crystalized with pyruvate bound. Upon protein structure overlay, the Zm-Pdc structure was hidden, leaving only the pyruvate, TPP analogue and Mg^2+^ and the Ll-KdcA active site shown. The pyruvate molecule served as an indicator of the putative binding position and orientation of the 2-ketoacid substrates within Ll-KdcA, after which the synthetic 2-keto-4 hydroxybutyrate (OHB) substrate was manually docked. The individual mutation at valine residue 461 was introduced to Ll-KdcA manually in Pymol and resulting change in pocket size is shown.

Manual docking of OHB into the active site pocket of Zm-Pdc revealed the substrate binding site to be too small for accommodation of this substrate (Fig. 3A). We speculated that the possible clash of the bulky tryptophan residue at position 392 located in the deep cleft leading to the active site with OHB can be avoided by replacement with glutamine (Fig. 3B), which may then be able to hydrogen bond with the 4-OH hydroxyl group. This W392Q mutation in Zm-Pdc resulted in a slight 1.4-fold increase of catalytic efficiency on OHB but also in nearly 100-fold reduction of its catalytic efficiency towards the natural substrate pyruvate (Table 2). This result suggested that it is very difficult to adapt the binding site of the Zm-Pdc larger ketoacid substrates. On the other hand, the substrate binding site in KdcA from *L*. *lactis* can accept a wide range and more voluminous molecules including branched chain substrates^25^. In particular, the residue Val461 (Fig. 4A) lies at the entrance of the substrate (*S*)-pocket to which it can control access. In addition, the active site of Ll-KdcA is lined with several other key residues previously identified to play a critical role in controlling substrate pocket size and acceptance of bulkier substrates, including Phe381, Gly402, Met538, and Phe542^25^. Replacement of valine at position 461 by a bulkier isoleucine residue is expected to further restrict the size of the *S*-pocket and to improve interactions with OHB in the main substrate channel (Fig. 4B). As reported in Table 2, the substitution of Val461 by isoleucine in Ll-KdcA indeed resulted in a 3-fold increase of V_max_ on OHB and in a 2-fold reduction of the K_m_ for this non-natural substrate.

### Production of 1,3-propanediol from DHB

We next set out to test the possibility of converting DHB to PDO using the DHB dehydrogenase, OHB decarboxylase and PDO oxidoreductase enzymes. For this purpose, we assembled an operon composed of genes coding for the DHB dehydrogenase Ec-LldD V108C variant, the endogenous aldehyde reductase YqhD and either one of the 4 different candidate OHB decarboxylases, namely Pdc, Pdc W382Q, KdcA, or KdcA V461I,. The operons were cloned into the medium-copy pACT3 vector yielding pPDO-1, pPDO-2, pPDO-3 and pPDO-4 (see Table 4). The resulting constructs were incubated on mineral medium that contained glucose as the carbon source and which was supplemented with 50 mM racemic (D/L)-DHB. PDO production and DHB consumption were estimated after 24h of cultivation. All strains, including the control strain, which only harbored an empty plasmid, consumed nearly identical amounts of DHB (Table 3). Furthermore, chromatographic separation of DHB stereoisomers revealed that mainly (L)-DHB was consumed by the bacterial cells. Since (L)-DHB is structurally similar to (L)-lactate, and since native Ec-LldD was shown to have activity on (L)-DHB we tested whether deletion of LldD in the wild-type strain could abolish DHB consumption. Indeed, we found that the Δ*lldD* mutant was unable of consuming DHB, thus, confirming the pivotal role of this enzyme in DHB assimilation (see Table 3).

**Table 4:** Plasmids constructed in this work.

Plasmid	Relevant characteristics	Source
pACT3	Cm^R^; p15A ori; promoter pTAC	^42^
pEXT20	Amp^R^; colE1 ori; promoter pTAC	^42^
pPDO-1	pACT3-llDV108c-kdcA-yqhD	This work
pPDO-2	pACT3-llDV108c-kdcAV461I-yqhD	This work
pPDO-3	pACT3-llDV108c-pdc-yqhD	This work
pPDO-4	pACT3-llDV108c-pdcW392Q-yqhD	This work
pPDO-5	pEXT20-llDV108c-pdcW392Q-yqhD	This work
pPDO-6	PEXT20-llDV108c-kdcAV461I-yqhD	This work

While wild-type cells were able of consuming DHB, PDO production was only observed in cells that expressed PDO pathway enzymes indicating that the proposed reaction sequence is feasible and that at least part of the required enzymatic activities are not naturally present in *E*. *coli*. In particular, the conversion rate of L-DHB into PDO was impacted by OHB decarboxylase enzyme. Strains expressing Ll-KdcA V461I (strain Pen913) and Zm-Pdc W392Q (strain Pen966) variants showed the highest PDO titers (5.5 and 7.1 mM, respectively). Notably, strain Pen966 was capable of converting all the (L)-DHB that was consumed into PDO, indicating that the *in vivo* OHB decarboxylase activity of the Zm-Pdc W392Q variant is higher than that of Ll-KdcA V461.

The finding that only half the DHB initially present in the incubation medium was consumed even after 2 or 3 days of incubation at 37 °C by the *E*. *coli* strains indicated that either the DHB uptake and/or DHB oxidation could restrict its assimilation. As a first step to evaluate this possibility, the PDO-producing strain Pen913 was incubated with increasing amounts of (D/L)-DHB (from 10 to 100 mM) and DHB consumed as well as PDO produced were determined after 24 h. The result of this experiment showed that the consumption actually increased with the increase of exogenous DHB concentration and reached a plateau at 50 mM, whereas PDO production steadily increased with the increase of DHB concentration (see Fig. S2 in Supplementary Information). This data supported the idea of a limitation by either the uptake or the oxidation of DHB. To distinguish between these two possibilities, we took into account the report of METEX^26^ which discussed the capacity of various permeases from *E*. *coli* and notably glycolate permease (GlcA) and α-ketoglutarate permease (KgtP) that may enhance DHB efflux. In line with this information, we investigated the effect of these aforementioned transporters on the DHB consumption in strains that expressed the DHB-PDO pathway. Interestingly, an increase of more than 2-fold in DHB consumption was observed only in strains that overexpressed *glcA* (Fig. S3 in Supplementary Information). In addition, under this condition, both (D)- and (L)- forms of DHB were consumed with 75% preference for (L)-DHB. While these results confirmed that the uptake of DHB is one of the limiting factors for its bioconversion, they also showed that part of the DHB consumed is accumulated either as an intermediate of the PDO pathway or has been assimilated by the cells by another pathway.

### Production of 1,3-propanediol from glucose

**Figure 5:**
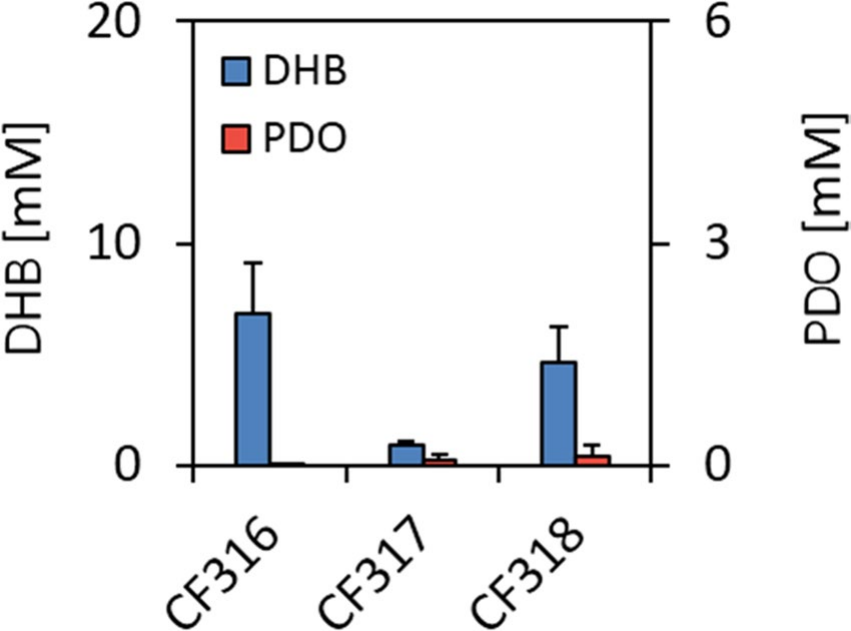
PDO production from glucose after 24 h of cultivation of an *E*. *coli* monoculture system. Cells were cultivated in 250 mL baffled shake flasks on mineral medium containing 20 g L^−1^ glucose. At OD_600_~0.6, IPTG (1 mM) was added to the medium. Reported data were the mean (±SD) of at least two replicates.

To demonstrate PDO production directly from glucose, we cloned the genes coding for the complete six-reaction steps pathway into two different plasmids. The previously constructed medium-copy pDHBop-ppc* vector provided all enzymatic activities required to transform malate into DHB by driving the expression of malate kinase (variant Ec-LysC V115A:E119S:E250K:E434V), malate semialdehyde dehydrogenase (variant Bs-Asd E218Q), and malate semialdehyde reductase (variant Ms-Ssr H39R:N43H) enzymes^19^. Overexpression of the malate-insensitive phosphoenolpyruvate carboxylase variant Ec-Ppc K620S from the pDHBop-ppc* plasmid was previously shown to greatly increase DHB production^19,27^. The three genes responsible for the conversion of DHB to PDO were cloned into a high-copy vector yielding pPDO-5 or pPDO-6 (see Table 4). These constructed plasmids were transformed into *E*. *coli* MG1655 strains yielding strain CF317 (pDHBop-ppc* and pPDO-5) and CF318 (pDHBop-ppc* and pPDO-6), which were cultivated for 24 h on mineral medium containing 20 g.L^−1^ glucose as the carbon source. Only the strains that expressed all pathway genes produced PDO albeit at a very low level, whereas the control strain CF316 which only harbored pDHBop-ppc* and the empty pEXT20 plasmid was incapable of PDO production (Fig. 5). While it is still unclear why DHB excretion was 5-fold higher with CF318 than with CF317, these results suggested that the very low PDO production from glucose could be associated to a weaker catalytic capacity of the downstream PDO pathway as compared to that of the DHB-yielding pathway.

**Figure 6:**
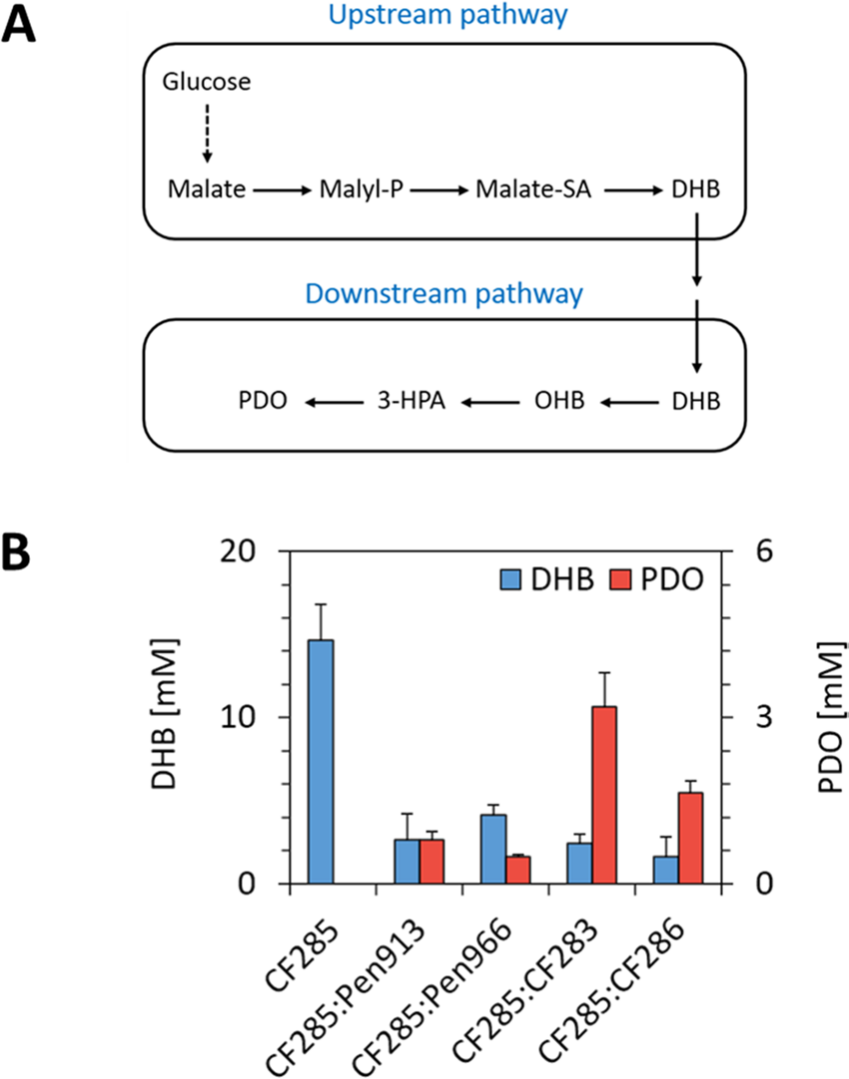
PDO production from glucose after 24 h with two *E*. *coli* strain co-culture systems. (**A**) Scheme of the co-culture design, in which a first cell expresses the upstream pathway enabling DHB synthesis from glucose, while a second cell incorporated extracellularly accumulated DHB and converts it to PDO due to expression of the downstream PDO producing pathway. (**B**) Production of PDO from glucose at inoculation ratio equal to 1:1 with four different PDO producing strains (Pen913, Pen966, CF283, CF286) and the DHB producing strain CF285.

Besides the fact that the pathway enzymes are yet not optimal, another reason for the very weak production of PDO from glucose could be due to the metabolic burden on the producing strain which has to carry out two plasmids (medium and high copy number)^28^. To evaluate this possibility, we considered following a co-cultivation strategy in which an *E*. *coli* strain expresses the synthetic DHB-yielding pathway while another one bears the PDO-producing pathway. Adopting such strategy was further supported by the previous demonstration of bacterial co-cultures as a mean towards increased production of small molecules, including muconic acid^29^, 3-amino-benzoic acid^30^ and *n*-butanol^31^. The design of our co-culture strategy is shown in Fig. 6A. The upstream DHB-yielding pathway was expressed from the CF285 strain whilst the downstream part of the pathway that converted DHB into PDO was expressed in the strains Pen913 or Pen966. To evaluate the performance of the co-cultivation strategy, the DHB- and PDO-producing strains were cultivated for 24h in a 1:1 ratio at the inoculation stage on glucose-containing mineral medium. In a control experiment, strain CF285 that only expresses the DHB-yielding pathway produced 14.7 mM DHB but no PDO, whereas the co-cultivation of this strain with Pen913 or Pen 966 resulted in a PDO production that was 5 to 8 fold higher than with a strain expressing both pathways (compared data in Fig. 5 with those in Fig. 6B). In both cases, DHB accumulated in the medium, which supported the idea that the PDO-producing pathway module is catalytically less efficient that the DHB-producing pathway module. Since the overexpression of *glcA* was found to enhance DHB uptake (see Fig. S3 in Supplementary Information), we repeated this co-cultivation strategy using strain CF283 (MG1655 *glcA*^proD^ bearing pPDO-2 or strain CF286 (MG1655 *glcA*^proD^ with pPDO-4). A PDO production of up to 4 mM was obtained with CF285:CF283 strain co-cultivation, whereas co-culture of CF285: CF286 produced only 1.5 mM PDO, confirming our earlier results (see above) that the OHB decarboxylase mutant Zm-Pdc W382Q was more efficient than the Ll-KdcA V461I for *in vivo* production PDO from glucose. In an attempt to increase PDO production, we doubled the amount of CF283 versus CF285 at the inoculation stage to compensate for the weaker activity of this pathway module. However, this change in the cultivation protocol did not improve PDO titers (data not shown). Altogether, these results demonstrate that the production of PDO directly from glucose is feasible and that, at least ate the present stage of the development, distributing pathway modules into two strains that are co-cultivated increases PDO titers.

## Discussion

In this work, we explored the feasibility of a synthetic pathway enabling PDO biosynthesis from glucose under aerobic conditions via the Krebs cycle intermediate (L)-malate, which is based on the extension of a previously published (L)-DHB metabolic pathway^19^. The synthetic route does not involve supplementation of the expensive vitamin-B12 or the use of glycerol as a precursor unlike naturally occurring PDO pathways, but it relies on NAD(P)H co-factor and ATP to achieve relevant PDO titers. In this scope, we recently engineered *E*. *coli* to produce malate via Krebs cycle and glyoxylate shunt, which is expected to provide additional ATP and NAD(P)H cofactors which are necessary to convert malate to DHB or PDO^32^. The theoretical yield of this Krebs cycle-dependent PDO production was calculated to be 1.5 mol PDO per mol glucose. Since this value is similar to other PDO pathways that proceed through glycerol^11,17^, the synthetic pathway presented here may be an alternative or complement for the industrially established PDO production routes.

One of the major challenges during the construction of non-natural pathways consists in finding and engineering the constituting enzymes to obtain activities that are compatible with in *vivo* applications. The proposed route uses a combination of natural and engineered enzymes acting on non-natural substrates. While the three first catalytic steps linking (L)-malate to DHB were constructed in a previous work^19^, the identification and engineering of enzymes catalyzing the three remaining enzymatic activities to convert DHB into PDO was targeted in this work. Based on a screening of candidate enzymes performing similar reactions and acting on sterically cognate substrates, enzymes with (L)-DHB dehydrogenase and OHB decarboxylase activities were identified. These enzymes exhibited, however, weak activities on the non-natural substrates. We therefore employed a rational enzyme engineering strategy which relied on the previously reported structural analysis of the 2-keto acid decarboxylase KdcA^25,33^, and the *Saccharomyces cerevisiae* mitochondrial lactate oxidoreductase, the homologous to *E*. *coli LldD*^34^. Although the increase of activity of these enzymes on the non-natural substrates by a rational engineering strategy gave some limited success, it also provided interesting insights about the impact of the OHB decarboxylase in the DHB to PDO conversion, since only *E*. *coli* strains expressing Zm-Pdc_W392Q_ variant together with LldD_V108C_ was able to convert almost all L-DHB into PDO. In the other cases, the conversion rate was much lower indicating that OHB obtained from the oxidation of DHB by the LldD enzyme has been diverted into other pathway(s). The most likely explanation is the conversion of OHB into homoserine, by mean of a transaminase reaction involving glutamate as the ‘NH_2_’ donor. This hypothesis is supported by the finding that we previously reported that aspartate aminotransferase encoded by aspC can catalyze the reversible reaction homoserine + α-ketoglutarate <−> 4-hydroxy-2-keto butyrate + glutamate^27^. Importantly, the direction of this reaction is depending on the glutamate/ α-ketoglutarate ratio, which is very high in *E*. *coli*^35^. Therefore, there seems to be a key competition at the level of OHB between a transaminase and a decarboxylase reaction. Generation and screening of larger mutant libraries appears to be required for obtaining OHB decarboxylase as well as DHB dehydrogenase enzymes with adequate activities to solve this issue and favor the DHB-PDO production pathway. In this regard, our recently developed transcription-based aldehyde sensor^36^, which also responded to the PDO pathway intermediate 3-hydroxypropanal, could be an exquisite tool for *in vivo* screening of more efficient OHB decarboxylases as well as enzyme(s) mutant libraries generated of upstream reaction steps in the pathway.

Even though the constituting enzymatic activities of the synthetic pathway were rather low, we could demonstrate a direct production of 0.1 mM PDO from 110 mM glucose by expression the whole pathway bearing six enzymatic reactions in a single *E*. *coli* strain. Moreover, we could show that this weak production was in part due the metabolic burden on the production strain, which had to carry two plasmids since a co-cultivation strategy where the pathway was split into two functional modules resulted in a 10-fold increase in PDO titer. Also, another limitation that was in part alleviated was to engineer a better uptake of the DHB through overexpression of the GlcA permease, which further increase to PDO production by 4-fold. While these results are in line with previous studies, which also reported increase production rates upon co-culturing strains that express only parts of a long metabolic pathway^30^, this strategy can only be applied at very early stages of process development. For an industrial application of this pathway it is not a preferable solution, since production of bulk chemicals such as PDO requires extremely high carbon yields that cannot be achieved by co-culturing two strains. Thus, obtaining pathway enzymes with higher activities and which will require their moderate overexpression to provide adequate *in vivo* activities is the key for constructing robust production strains that have industrially relevant PDO production rates.

## Methods

### Chemicals and reagents

All chemicals and solvents were purchased from Sigma-Aldrich unless otherwise stated. Restriction endonucleases and DNA-modifying enzymes were from New England Biolabs and used according to manufacturer’s instructions. DNA plasmid isolation was performed using GeneJET Plasmid Miniprep Kit (Thermo Scientific). DNA extraction from agarose gel was carried out using the GeneJET Gel Extraction Kit (Thermo Scientific). DNA sequencing was carried out by Beckman Coulter Genomics (Takeley, United Kingdom) or Eurofins SAS (Ebersberg, Germany). The racemic mixture of sodium (D/L)-DHB (purity, 70%) was chemically synthetized by Adisseo SA (France).

### Protein cloning and mutagenesis

*E*. *coli* DH5α (New England Biolabs) was routinely used for construction of plasmids. Wild-type Ec-*lldD* and Zm-*pdc* genes were amplified from genomic DNA (extracted from *E*. *coli* MG1655 and *Zymomonas mobilis* ATCC^®^ 31821, respectively) by PCR. The used primers are listed in Table S2. The gene Ll-*kdcA* from *Lactococcus lactis* B1157-NIZO was codon-optimized for expression in *E*. *coli* and synthesized by Eurofins. The resulting DNA fragments were digested with suitable restriction enzymes (Table S2) and cloned into the corresponding sites of pET28a vector system (Novagen) using T4 DNA ligase (Biolabs), thereby adding an N-terminal hexa-His tag. Point mutations were introduced on pET28-derived plasmid by inverse PCR using the primers listed in Table S3. Resulting products were digested by DpnI to remove template DNA and transformed into competent cells. The introduction of desired mutation was verified by sequencing.

### Protein expression and purification

Enzymes were expressed in *E*. *coli* BL21 (DE3) cells (New England Biolabs) in 200 mL Luria-Bertani (LB) medium supplemented with 50 µg/mL kanamycin (37 °C, 200 rpm). Expression cultures were inoculated from an overnight culture at OD_600_ of 0.05 and grown to OD_600_ of 0.6 before protein expression was induced by addition of 1 mM isopropyl β-D-1-thiogalactopyranoside (IPTG) to the culture medium. Cells were harvested after 3 h of incubation by centrifugation (15 min at 4,000 rpm, 4 °C) and pellets stored at −20 °C until further analysis. Frozen cell pellets were resuspended in 1 mL of lysis buffer (50 mM Hepes, 300 mM NaCl, pH 7.5) and disrupted by four successive rounds of sonication (sonication interval: 30 s, power output: 30%, sonicator: Bioblock Scientific, VibraCell™ 72437). The resulting cell crude extract was directly used for measurement of FMN-dependent 2-hydroxyacid dehydrogenase activities, while 2-ketoacid decarboxylase enzymes were purified as described elsewhere^19^.

### Enzymatic assays

The activity of wild-type membrane-associated (L)-lactate dehydrogenase and mutant variant V108C assayed in the oxidative direction by monitoring reduction of 2,6-dichloroindophenol (DCIP) at 655 nm (ε = 5.9 mM^−1^ cm^−1^) during oxidation of 2-hydroxyacids. The assay mixture contained 60 mM Hepes (pH 7), 50 mM KCl, 0.06 mM DCIP and appropriate amounts of crude protein extract. Reactions were started by adding appropriate concentrations of (L)-lactate or a racemic mixture of (D/L)-DHB (Adisseo SAS, France). One unit of membrane-associated dehydrogenase activity (U) was defined as the amount of enzyme catalyzing the conversion of 1.0 µmole of DCIP per minute.

The activity of keto-acid decarboxylase wild-type and mutant variants was assayed by coupling the decarboxylation reactions to the NAD(P)H-dependent reduction of the produced aldehydes. The decarboxylation of natural substrates (pyruvate, 3-methyl-2-oxobutyric acid) was coupled to the NADH-dependent reduction of acetaldehyde catalyzed by yeast alcohol dehydrogenase (Sigma, A7011). OHB decarboxylase activity was assayed by coupling decarboxylase activity to the NADPH-dependent reduction of the released 3-hydroxypropanal by purified aldehyde reductase Ec-YqhD. The assay mixture contained 60 mM Hepes (pH 7), 50 mM KCl, 5 mM MgCl_2_, 0.25 mM NAD(P)H, 0.5 mM thiamine pyrophosphate, 100 µg mL^−1^ auxiliary enzyme and appropriate amounts of purified enzyme. Reactions were started by adding appropriate concentrations of pyruvate, 3-methyl-2-oxobutyric acid or OHB. The latter was synthesized in-house as previously described^27^. One unit of decarboxylase activity (U) was defined as the amount of enzyme catalyzing the conversion of 1.0 µmole of 2-ketoacid per minute

All enzyme assays were performed in a microplate reader (Epoch 2, BioTek) at 37 °C in 96-well flat-bottomed microtiter plates in a final volume of 250 µL. Protein concentrations were determined prior to enzymatic assays by Bradford method^37^.

### Plasmids and strains construction

Plasmids and strains that were constructed and used in this study are listed in Tables 4 and 5. The gene *yqhD* was amplified by PCR from genomic DNA with primer pairs pen290/pen291, whilst the remaining genes were amplified from pET28-28 derived vectors using primer pairs listed in Table S4. DNA fragments were purified and assembled by homologous recombination with BamHI/SalI digested pEXT20 or pACT3 vector using the NEBuilder® HiFi DNA Assembly kit (New England Biolabs). The resulting plasmids were transformed into DH5α competent E. coli cells and assembled operons verified by DNA sequencing.

**Table 5:** Strains used in this work.

Strain	Genotype	Source
NEB5-α	*E. coli fhuA2 Δ(argF-lacZ)U169 phoA glnV44 Φ80Δ (lacZ)M15 gyrA96 recA1 relA1 endA1 thi-1 hsdR17*	NEB
BL21 (DE3)	*E. coli fhuA2 [lon] ompT gal* (*λ DE3*) *[dcm]* ∆*hsdS*	NEB
MG1655	*E. coli* F^−^λ^−^ ilvG-rfb-50 rph-1	ATCC 47076
CF220	MG1655 *glcA*^proD^	This work
CF221	MG1655 *kgtP*^proD^	This work
Pen946	MG1655/pACT3	This work
Pen911	MG1655/pPDO-1	This work
Pen913	MG1655/pPDO-2	This work
Pen965	MG1655/pPDO-3	This work
Pen966	MG1655/pPDO-4	This work
CF285	MG1655/pDHB_op_ (ppc*)	^19^
CF316	CF285/pEXT20	This work
CF317	CF285/pPD0-5	This work
CF318	CF285/pPDO-6	This work
CF283	CF220/pPDO-2	This work
CF284	CF221/pPDO-2	This work
CF286	CF220/pPDO-4	This work

*E*. *coli* K-12 substr. MG1655 (ATCC 47076) was used as the parental strain for all constructions in this study (Table 5). Expression of *glcA* and *ktgP* genes was rendered constitutive by replacing the native chromosomal 5′-UTR of each gene by the synthetic constitutive and insulated promoter proD^38^. The proD sequence was preceded by a kan resistance cassette which was amplified by PCR adding 50 bp flanking sequences that were homologous to the target locus. The resulting DNA fragment was used to replace the natural gene promoter by homologous recombination^39^. Primers used are listed in Table S4. Positive clones were selected on LB agar plates containing kanamycin (50 µg mL^−1^) and verified by PCR analysis. The kan cassette was removed from the genome by expressing FLP recombinase from the pCP20 plasmid^40^ and correct excision of the cassette was verified by PCR using locus specific primers (Table S4). Plasmids were transformed into the target *E*. *coli* strains using standard laboratory protocols^41^.

### Condition for PDO production from DHB or Glucose

All cell cultivation was carried out at 37 °C on a rotary shaker (Infors HT, France) running at 200 rpm. Pre-cultures were grown in 5 mL of LB in 50 mL falcon tubes. After ~10 h, 500 µL were used to inoculate a second pre-culture (10 mL of 90% v/v M9 mineral medium supplemented with 20 g L^−1^ glucose and 10% v/v LB in 50 mL falcon tubes) that was cultivated overnight. The biomass needed to start main cultures with a starting OD_600_ of 0.2 was transferred to 250 mL baffled shake flasks containing 25 mL of 90% v/v M9 mineral medium supplemented with 20 g L^−1^ glucose and 10% v/v LB, and 1 mM IPTG and 50 mM (D/L)-DHB were added when OD_600_ reached ~0.6. The antibiotic chloramphenicol was added when required at 25 mg L^−1^. M9 mineral medium contained: 20 g glucose, 18 g Na_2_HPO_4_*12H_2_O, 3 g KH_2_PO_4_, 0.5 g NaCl, 2 g NH_4_Cl, 0.5 g MgSO_4_*7H_2_O, 0.015 CaCl_2_*2H_2_O, 1 ml of 0.06 M FeCl_3_ stock solution prepared in 100 times diluted concentrated HCl, 2 ml of 10 mM thiamine HCl stock solution, 20 g MOPS, and 1 ml of trace element solution (containing per liter: 0.04 g Na_2_EDTA*2H_2_O, 0.18 g CoCl_2_*6H_2_O, ZnSO_4_*7H_2_O, 0.04 g Na_2_MoO_4_*2H_2_O, 0.01 g H_3_BO_3_, 0.12 g MnSO_4_*H_2_O, 0.12 g CuCl_2_*H_2_O). The pH was adjusted to 7.0 and the medium was filter-sterilized.

Pre-culture was made in 5 mL of LB in 50 mL falcon tubes. After ~10 h, 500 µL were used to inoculate a second pre-culture (10 mL of M9 mineral medium supplemented with 20 g L^−1^ glucose in 50 mL falcon tubes) that was cultivated overnight. The biomass needed to start main cultures with a starting OD_600_ of 0.2 was transferred to 250 mL baffled shake flasks containing 25 mL of M9 mineral medium supplemented with 20 g L^−1^ glucose. The antibiotics ampicillin, kanamycin sulphate and chloramphenicol were added when required, respectively, at 100, 50 and 25 mg L^−1^. IPTG (1 mM) was added after 3 h of cell cultivation. In co-cultivation experiments, the different strains were inoculated at a ratio described in the text.

### Analytical methods

Extracellular concentrations of metabolites were determined on a Dionex Ultimate 3,000 HPLC system (Thermo Scientific, France) equipped with a RI detector (RID-10A, Shimadzu, Japan) and UV/Vis detector (SPD-20A, Shimadzu). The sample injection volume was 20 μL. Glucose and (D/L)-DHB were measured with the RI detector by using a cation-exchange column (Rezex RoA-organic acid H^+^ 8%) preceded by a SecurityGuard guard cartridge (Phenomenex, USA). The separation was performed at 80 °C with 0.5 mM H_2_SO_4_ at 0.5 mL min^−1^ as mobile phase. PDO concentrations (from RI detector) were measured using an Aminex HPX-87H column protected by a Micro-Guard Cation H^+^ pre-column (BioRad, USA). The separation was performed at 35 °C with 1.25 mM H_2_SO_4_ at 0.5 mL min^−1^. Amounts of D-DHB were estimated on the UV detector (at 254 nm) by using a Chirex 3126 column (Phenomenex, USA) with an aqueous mobile phase containing 2 mM CuSO_4_ and 15% methanol. Flow rate was 1 mL/min, and the column was held at 22 °C. Concentrations of (L)-DHB were estimated by subtracting estimated amounts of the (D)-form to total (D/L)-DHB. All samples were centrifuged (2 min at 13,000 rpm) and syringe-filtered (0.2 µm), and the resulting supernatant stored at −20 °C before analysis. A standard calibration curve was obtained by injecting standards.

## Supplementary information

Supplementary info
