# Supplementary material for: Construction of a synthetic pathway for the production of 1,3-propanediol from glucose
Source: Sci Rep. 2019 Aug 9;9:11576. doi: 10.1038/s41598-019-48091-7 (PMC6689062; doi:10.1038/s41598-019-48091-7)
Supplement: Supplementary file 1 — Supplementary info [file 41598_2019_48091_MOESM1_ESM.pdf]

Supplementary information

Construction of a synthetic pathway for the production of 1,3-propanediol from glucose

Authors

Cláudio J. R. Frazão[$,1], Débora Trichez[$,1], Hélène Serrano-Bataille[1], Adilia Dagkesamanskaia[1], Christopher M. Topham[2], Thomas Walther[1,3 ; p§] and Jean Marie François[1,3*]

[1]LISBP, Université de Toulouse, CNRS, INRA, INSA, 135 Avenue de Rangueil, F-31077 Toulouse, France

[2]Molecular Forces Consulting, 40 rue Boyssonne, F-31400 Toulouse, France

[3]TWB, 3 Rue des Satellites, Canal Biotech Building 2, F-31400 Toulouse, France

[$]These authors contributed equally to this work

[§]Present address: TU Dresden, Institute of Natural Materials Technology, 01062 Dresden, Germany

*Correspondence to: Jean Marie Francois : email fran_jm@insa-toulouse.fr; ph : +33 (0) 61 55 94 92

Table S1. Gibbs free energy of formation of PDO pathway intermediates.

**Compound**	**Contributing groups**	**$\Delta_f G^{0\prime}$ [kJ mol^{-1}]**
malate^{2-}	2x –COO^{1-}<br>1x -OH<br>1x -CH_2-<br>1x –CH$<$	-840.98
malyl-P^{2-}	1x –COO^{1-}<br>1x -OH<br>1x -CH_2-<br>1x –CH$<$<br>1x -CO-OPO_3H^-	-1740.1
malate-SA$^-$	1x -COO^{1-}<br>1x –OH<br>1x -COH<br>1x -CH_2-<br>1x –CH$<$	-620.5
DHB$^-$	1x -COO^{1-}<br>2x –OH<br>2x -CH_2-<br>1x –CH$<$	-660.2
OHB$^-$	1x -COO^{1-}<br>1x –OH<br>2x -CH_2-<br>1x $>$CO	-626.6
3-HPA	1x –OH<br>2x -CH_2-<br>1x $>$CO	-287.3
PDO	2x –OH<br>3x -CH_2-	-326.9
ATP3-<br>ADP2-<br>NADPH<br>NADP+<br>HPO42-<br>H+<br>NADH<br>NAD+<br>FMN<br>FMNH2<br>CO2		-2819.4<br>-1949.1<br>-3082.9<br>-3104.9<br>-1096.1<br>-39.9<br>-2193.8<br>-2215.8<br>-1352.4<br>-1449.0<br>-386.0

Table S2. Primers and restriction enzymes used to clone genes into pET28a expression vector

Gene*	Primer sequences (5' – 3')	Restriction enzymes	Resulting vector
Ec-*lldD*	CATATGATTATTTCCGCAGCCAGC<br><br>AGATCTCTATGCCGCATTCCCTTTC	NdeI<br>BgI2	pET28-Ec-lldD
Zm-*pdc*	CATATGAGTTATACTGTCGGTACC<br><br>GGATCCCTAGAGGAGCTTGTTAAC	NdeI<br>BamHI	pET28-Zm-pdc
Ll-*kdcA*	provided in vector by Eurofins	NheI<br>EcoRI	pET28-Ll-kdcA

* Ec (from *Escherichia coli),* Zm (from *Zymomonas mobilis)*, Ll (from *Lactococcus lactis)*

Table S3. Primers used for site-directed mutagenesis

Matrix	Mutated position	Primer sequences (5' – 3')	Restr. site
pET28-Ec-lldD	V108C	TTCCGTTTACTCTGTCGACGTGTTCCGTTTGCCCGA<br><br>TCGGGCAAACGGAACCCGTCGACAGAGTAAACGGAA	HincI<br>I
pET28-Zm-Pdc	W392Q	GTTATTGCTGAAACCGGTGACTCTCAGTTCAATGCGCAGCG<br>CATGAAGC<br><br>GCTTCATGCGCTGCGCATTGAACTGAGAGTCACCGGTTTCA<br>GCAATAAC	FspI
pET28-Ll-kdcA	V461I	TTTGCTTTATCATTAATAATGACGGCTACACAATCGAGCGC<br>GAAATTCA<br><br>TGAATTTCGCGCTCGATTGTGTAGCCGTCATTATTAATGATA<br>AAGCAAA	AseI

Table S4. Primers used in strain construction.

Primer	DNA sequences (5' – 3')
Plasmid construction	
pen286 (fw-IldD)	CGGTACCCGGGGATCCTGCCCTAGCCTATTCGATTAAGGAGGTCAAATATGATTATTTCCGCAG CCAG
pen287 (rv-IldD-kdcA)	TTGGTGTTATTTTTGACTATGCCGCATTCCCTTTCGC
pen288 (fw-kdcA)	TCAAAAATAACACCAAATCAATAAGGAGGAACGTTAtgtataccgttggggattatc
pen289 (rv-kdcA)	TCCTTAGTGGATATCGttatttgttctgttcagcaaac
pen290 (fw-yqhD)	CGATATCCACTAAGGAGGTAACTAATGAACAACTTTAATCTGCA
pen291 (rv-yqhD)	ATGCCTGCAGGTCGACTTAGCGGGCGGCTTCGTATA
pen292 (rv-lldD-pdc)	ATATGTTTTATCTGATCTATGCCGCATTCCCTTTCGC
pen293 (fw-pdc)	ATCAGATAAAACATATTTTAGAGGAGGTAGTAAATGAGTTATACTGTCGGTACC
pen294 (rv-pdc)	TCCTTAGTGGATATCGTTATTTGTTCTGTTCAGCAAAC
Strain construction	
cf245 (kgtP-prod_fw)	ATGGTTAAGGTTGCATAATGATATGCAACAAATGTATAATATTTCGTGTAGGCTGGAGCTGCTTC
cf246 (kgtP-prod_rv)	GTATCACTACTTGTCAGTTTGCTGTCTGCCGTTACAGTACTTTCAGCcatATAATACCTCCTAAAGTT AAACAAAATTATTTGTAG
cf253 (glca-prod_fw)	CCGAACCGTTATTACACGCCTGGCGTTTACGCGAAAAAGAAAGTCATTAAGTGTAGGCTGGAGC TGCTTC
cf254 (glca-prod_rv)	GCCCCAGTCCTCCCATCGGCATATACATTTGGGTCCAGGTAACcatATAATACCTCCTAAAGTTAAA CAAAATTATTTGTAG
Verification primers	
cf247 (kgtP_ver_fw)	ATACGTGTCC TCCTTACCAG
cf248 (kgtP_ver_rv)	AGTAGAGTGAACAGAACGAGTA
cf255 (glcA_ver_fw)	AATTTCGCTA ACTCGTG
cf256 (glcA_ver_rv)	ATGTCCTTTCAGACGTAATA

Note 1: Standard Gibbs free energy of the DHB pathway

The stoichiometry of the reactions malate kinase (MK), malate semialdehyde dehydrogenase (MSD) and malate semialdehyde reductase (MSR), 2,4-dihydroxybutyrate dehydrogenase (DD), OHB decarboxylase (OD) and aldehyde reductase (AR) is as follows (Equations S1 – S6):

MK: $malate^{2-} + ATP^{3-} + H^+ = malyl\text{-}P^{2-} + ADP^{2-}$ (S1)

MSD: $malyl\text{-}P^{2-} + NADPH = malate\text{-}SA^- + NADP^+ + HPO_4^{2-}$ (S2)

MSR: $malate\text{-}SA^- + NADPH + H^+ = DHB^- + NADP^+$ (S3)

DD: $DHB^- + FMN + H^+ = OHB^- + FMNH_2$ (S4)

OD: $OHB^- + H^+ = 3\text{-}HPA + CO_2$ (S5)

AR: $3\text{-}HPA + NADPH + H^+ = PDO + NADP^+$ (S6)

The standard Gibbs free energy for the formation of the DHB pathway intermediates ($\Delta_f G^{0'}$) was calculated based on the group contribution theory[1] using the dataset published by Jankowski[2] (Supplementary Table S1).

The standard Gibbs free energy of a reaction ($\Delta_r G^{0'}$) can be estimated according to Equation S8 from the standard Gibbs free energy of the formation of the participating compounds ($\Delta_f G^{0'}$), with *vi* being the stoichiometric coefficients.

$$\Delta_r G^{0'} = \sum_i \nu_i \cdot \Delta_f G_i^{0'} \quad \text{(S8)}$$

Accordingly, the standard Gibbs free energy of the reactions S1 – S6 are MK: 11 kJ mol^{-1}, MSD: 1.5 kJ mol^{-1}, MSR: -21.8 kJ mol^{-1}, DD: -23.1 kJ mol^{-1}, OD: -6.8 kJ mol^{-1}; AR: -21.7 kJ mol^{-1}. The upstream DHB pathway has a standard Gibbs free energy of -9.3 kJ mol^{-1}, while downstream PDO pathway displays a standard Gibbs free energy of -51.6 kJ mol^{-1}.

The complete PDO pathway has a standard Gibbs free energy of -60.9 kJ mol^{-1}.

Note 2: Calculation of the theoretical PDO yield

A previously published stoichiometric model of the central carbon metabolism in *E. coli*[3] was extended by 6 reactions steps enabling PDO production from malate. The theoretical yield was calculated based on elementary mode analysis using the CellNetAnalyzer software package[4]. The flux map showing one of the predicted carbon flux distributions that provide maximum yield was determined (data not shown). In the absence of cell growth, the maximum PDO yield is 1.5 mol per mol glucose.

Figure S1. Optimal carbon flux distribution for the production of 1,3-propanediol (PDO) in the central metabolic network of *E. coli*.

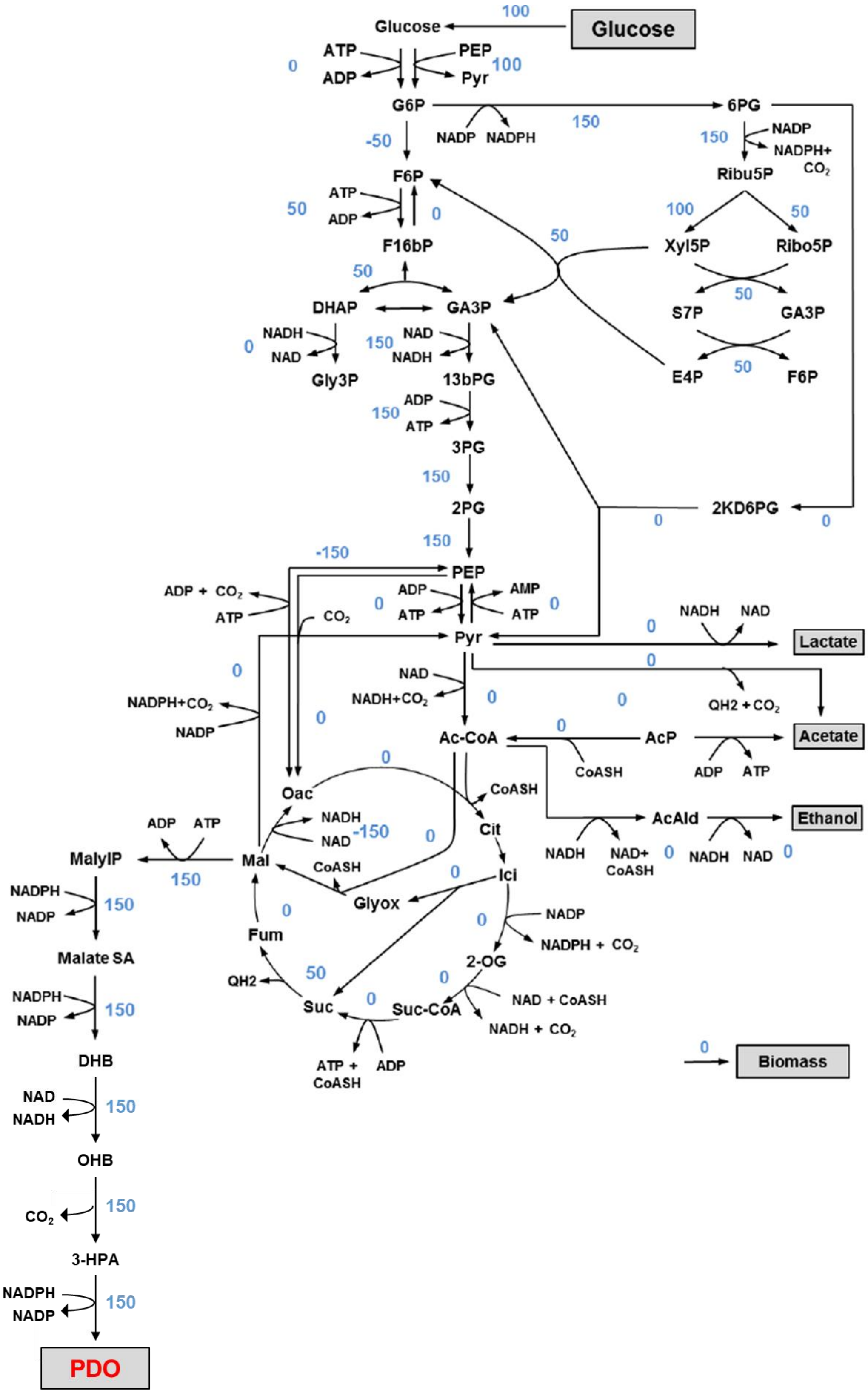

Figure S2. Influence of (D/L)-DHB feeding on PDO biosynthesis.

Production of PDO from 50 mM (D/L)-DHB after 24 h of culture of Pen913 strain (*E. coli* MG1655 pACT3-llDd$_{V108C}$-kdcA$_{V461I}$-yqhD). The error bars represent the standard deviation (SD) from the mean of two biological replicate experiments.

All cell cultivation was carried out at 37 ºC on a rotary shaker (Infors HT, France) running at 200 rpm. Pre-cultures were made in 5 mL of LB in 50 mL falcon tubes. After ~10 h, 500 µL was used to inoculate a second pre-culture (10 mL of 90 % v/v M9 mineral medium supplemented with 20 g L^{-1} glucose and 10 % v/v LB in 50 mL falcon tubes) that was cultivated overnight. The biomass needed to start main cultures with a starting OD_{600} of 0.2 was transferred to 250 mL baffled shake flasks containing 25 mL of 90 % v/v M9 mineral medium supplemented with 20 g L^{-1} glucose and 10 % v/v LB, and 1 mM IPTG and 50 mM (D/L)-DHB were added when OD_{600} reached ~0.6. The antibiotic chloramphenicol was added when required at 25 mg L^{-1}.

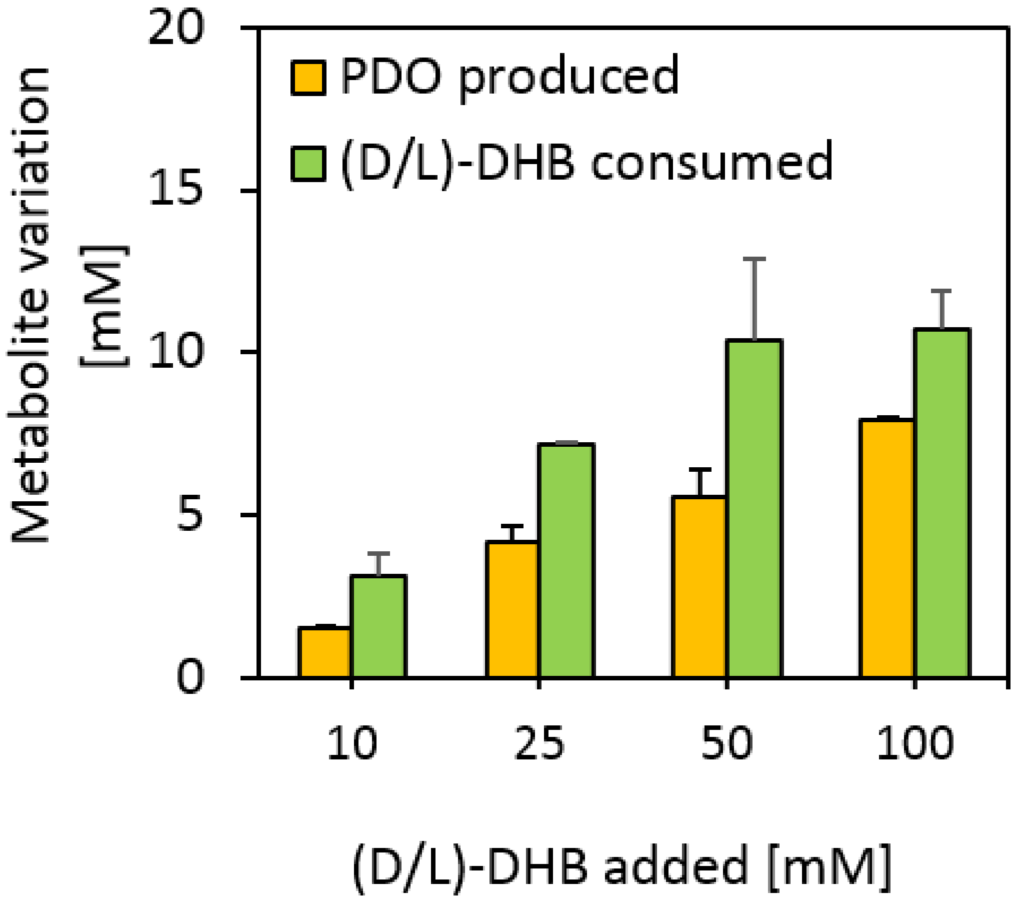

Figure S3. Influence of host strain on bioconversion of (D/L)-DHB into PDO.

Production of PDO from 50 mM (D/L)-DHB after 48 h of culture of various *E. coli* host strains harboring DHB downstream pathway encoded from the medium-copy plasmid pACT3-llDd$_{V108C}$-kdcA$_{V461I}$-yqhD. The error bars represent the standard deviation (SD) from the mean of two biological replicate experiments. All host strains originated from the parent strain *E. coli* MG1655. Conditions of cell cultivation are as in Figure S1

For each condition PDO yields on (D/L)-DHB were as follows, wt: 0.55 ±0.24 mol mol^{-1}, *glcA*^{proD}: 0.44 ±0.13 mol mol^{-1}, and *kgtP*^{proD}: 0.46 ±0.19 mol mol^{-1}.

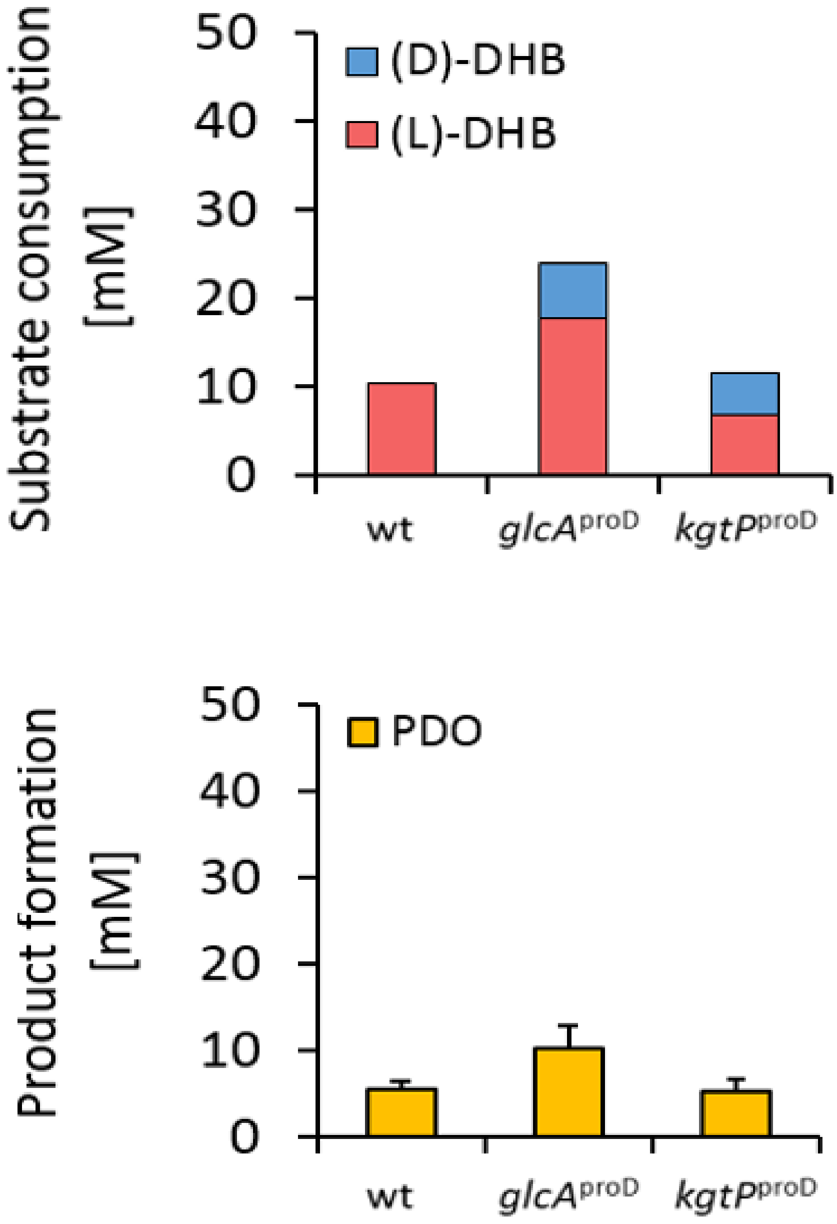

References.

1 Mavrovouniotis, M. L. Estimation of standard Gibbs energy changes of biotransformations. *J Biol Chem* **266**, 14440-14445 (1991).

2 Jankowski, M. D., Henry, C. S., Broadbelt, L. J. & Hatzimanikatis, V. Group contribution method for thermodynamic analysis of complex metabolic networks. *Biophys J* **95**, 1487-1499, doi:10.1529/biophysj.107.124784 (2008).

3 Stelling, J., Klamt, S., Bettenbrock, K., Schuster, S. & Gilles, E. D. Metabolic network structure determines key aspects of functionality and regulation. *Nature* **420**, 190-193, doi:10.1038/nature01166 (2002).

4 von Kamp, A., Thiele, S., Hadicke, O. & Klamt, S. Use of CellNetAnalyzer in biotechnology and metabolic engineering. *J Biotechnol* **261**, 221-228, doi:10.1016/j.jbiotec.2017.05.001 (2017).
